# Transcriptomes reveal the genetic mechanisms underlying ionic regulatory adaptations to salt in the crab-eating frog

**DOI:** 10.1038/srep17551

**Published:** 2015-12-01

**Authors:** Yong Shao, Li-Jun Wang, Li Zhong, Mei-Ling Hong, Hong-Man Chen, Robert W. Murphy, Dong-Dong Wu, Ya-Ping Zhang, Jing Che

**Affiliations:** 1 State Key Laboratory of Genetic Resources and Evolution, and Yunnan Laboratory of Molecular Biology of Domestic Animals, Kunming Institute of Zoology, Chinese Academy of Sciences, Kunming 650204, China; 2 Laboratory for Conservation and Utilization of Bio-resources, Yunnan University, Kunming 650091, China; 3 College of Life Sciences, Hainan Normal University, Haikou 571158, China; 4 Centre for Biodiversity and Conservation Biology, Royal Ontario Museum, 100 Queen’s Park, Toronto, Ont., M5S2C6, Canada; 5 Kunming College of Life Science, University of the Chinese Academy of Sciences, Kunming 650204, China

## Abstract

The crab-eating frog, *Fejervarya cancrivora*, is the only frog that lives near seas. It tolerates increased environmental concentrations of sodium, chloride and potassium partly by raising ion and urea levels in its blood plasma. The molecular mechanism of the adaptation remains rarely documented. Herein, we analyze transcriptomes of the crab-eating frog and its closely related saline-intolerant species, *F. limnocharis*, to explore the molecular basis of adaptations to such extreme environmental conditions. Analyses reveal the potential genetic mechanism underlying the adaptation to salinity for the crab-eating frog. Genes in categories associated with ion transport appear to have evolved rapidly in *F. cancrivora*. Both positively selected and differentially expressed genes exhibit enrichment in the GO category regulation of renal sodium excretion. In this category, the positively selected sites of *ANPEP* and *AVPR2* encode CD13 and V2 receptors, respectively; they fall precisely on conserved domains. More differentially expressed rapidly evolved genes occur in the kidney of *F. cancrivora* than in *F. limnocharis*. Four genes involved in the regulation of body fluid levels show signs of positive selection and increased expression. Significant up-regulation occurs in several genes of *F. cancrivora* associated with renin-angiotensin system and aldosterone-regulated sodium reabsorption pathways, which relate to osmotic regulation.

Most amphibians cannot survive in environments of greater than 10‰ salinity and no amphibian can survive permanently in seawater^1^. The crab-eating frog (*Fejervarya cancrivora*) is an exception to this tendency. It lives abundantly in coastal lowland areas and mangrove swamps of Southeast Asia where it tolerates a maximum of ~2/3 seawater and can survive in 75% or higher concentrations^2^. This resistance to hyperosmotic external environmental conditions generally owes to two physiological pathways: elevation of concentrations of salinity ions, especially sodium and chloride, in its blood plasma^3^ and accumulation of organic osmolyte-urea in tissues such as plasma and muscle via up-regulating hepatic urea synthesis^2^. Tadpoles of the crab-eating frog can better tolerate salinity than adults, but their osmotic regulation may be more similar to euryhaline teleosts^4^. The Eurasian green toad (*Pseudepidalea viridis*)^5^, the cane toad (*Rhinella marinus*)^6^ and at least one salamander (*Ambystoma tigrinum*)^7^,^8^,^9^ are also salt-tolerant. These lineages and the crab-eating frog may have functionally convergent genes^10^.

Amphibians use several hormonal systems to deal with salinity, including the hypothalamo–neurohypophysial, renin–angiotensin–aldosterone, adrenocorticotropic hormone–corticosteroid and natriuretic peptides systems^11^. Hormones of these systems can bind themselves to plasma membrane receptors by coupling with specific G proteins or cytosolic receptors to produce physiological effects^11^. Several amphibians increase the blood plasma parameters and concentrations of these hormones. For example, plasma osmolality in the cane toad correlates significantly with the concentration of arginine vasotocin^12^. Crab-eating frogs acclimated to hyperosmotic seawater elevate significantly their mean plasma concentrations of angiotensin II and aldosterone. Mean concentrations of plasma arginine vasotocin in dehydrated frogs were approximately 2.0 ~ 3.5 times higher than in control frogs^13^. In amphibians, aldosterone and corticosterone can increase sodium transport in organs involved in osmotic regulation, including skin, urinary bladder, and kidney^14^,^15^. Thus, these hormones appear to play vital roles in amphibian osmoregulation.

Osmotic regulation of euryhaline amphibians is a complicated process that requires further exploration, especially into the molecular basis of this adaptation. Prior studies have concentrated on physiological experiments, including measuring concentrations of plasma salinity ions and different hormones^1^,^3^,^13^. The expression levels of some proteins also have been investigated^2^. Complete gene expression profiles involved in high-salinity adaptations in the crab-eating frog remain undocumented.

High-throughput expression profiling technologies (RNA-seq) have altered our visions of the extent and complexity of eukaryotic transcriptomes in species ranging from yeast to humans^16^. This technology facilitates investigations into the genetic bases of adaptations. It has the potential to yield insights into the genetic adaptations of the crab-eating frog to its saline environment. This approach is especially valuable for non-model species that do not have a reference genome.

Herein, we elucidate the genetic adaptations of the crab-eating frog to its saline environment. *Fejervarya cancrivora* and *F. limnocharis* are morphologically similar^4^ sister species^17^,^18^,^19^. Whereas the former is saline-tolerant the latter is saline-intolerant, as is the closely related out-group species *Hoplobatrachus rugulosus*^17^,^18^,^19^. These associations provide the basis for understanding adaptations to living in brackish water. Multi-tissue transcriptomes for these frogs yield highly confident orthologous gene pairs and produce a comprehensive landscape of expression profiles for candidate genes involved in the adaptation. Network maps of positively selected and differentially expressed genes uncover the possible genetic base of the adaptation.

## Results

### *De novo* assembly and annotation of transcriptomes

RNA-sequencing generated 982.42 million raw reads involving about 98.24 Gb of data for brain, kidney and ventral skin tissues from *F. cancrivora*, *F. limnocharis* and *H. rugulosus*. After quality control, ~15.79 Gb of data per species were used for the *de novo* assembly of transcriptomes. In total, 188565 transcripts were assembled for *F. cancrivora*, 180476 for *F. limnocharis*, and 213730 for *H. rugulosus*. Our Contig N50s of all samples generally ranged from 955 bp to 1689 bp (Dataset 1). Thus, the quality of our *de novo* assemblies was similar to those of previous studies^20^,^21^.

**Figure 1:**
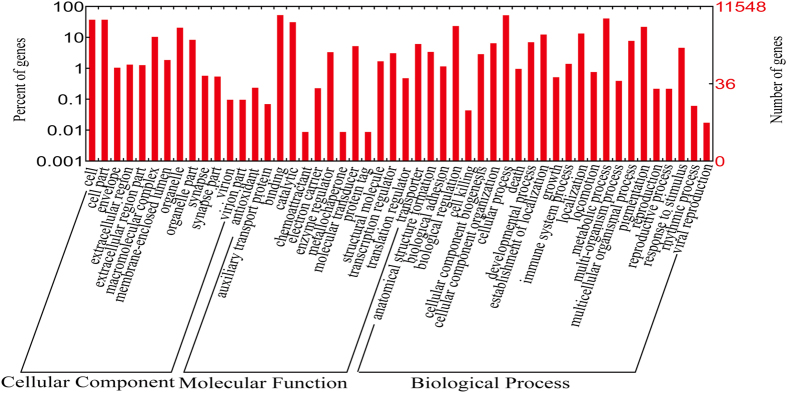
GO classification of annotated transcriptomic genes in *Fejervarya cancrivora*. Three levels (Biological Process, Molecular Function and Cellular Component) were demonstrated.

For *F. cancrivora*, 188565 transcripts with the largest contig N50s were annotated by *Blastx* against the protein database of the draft genome of *Nanorana parkeri*^22^. In total, 65312 contigs matched 16459 genes and these covered 75.0% of the protein-coding genes (21938) of *N. parkeri* (Dataset 2). The transcripts were also compared to the protein database of more distantly related *Xenopus tropicalis* (Ensembl, JGI_4.2.75) by using *Blastx*. A total of 57795 contigs of *F. cancrivora* matched 14106 protein-coding genes (76.5%) of *X*. *tropicalis* (Dataset 3). In total, 11548 protein-coding genes (81.9%) mapped to GO categories. We identified 55 main GO categories in three levels (23 GO terms in Biological Process, 14 GO terms in Molecular Function and 18 GO terms in Cellular Component) (Fig. 1 and Dataset 4) using *WEGO*^23^, a web tool for plotting GO annotations.

### Rapid evolution of coding sequences in *F*. *cancrivora*

**Figure 2:**
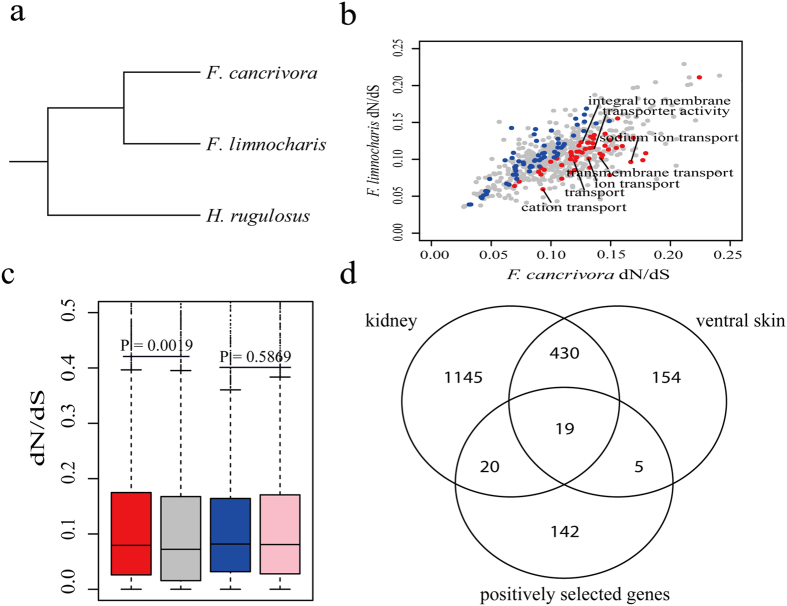
Evolutionary analyses of *Fejervarya cancrivora* and its closely related species, *F*. *limnocharis*. (**a**) The species-tree. (**b**) Comparison of dN/dS ratios between *F*. *cancrivora* and *F*. *limnocharis* by GO functional categories. Blue and red dots represent categories with an elevated evolutionary rates in *F*. *limnocharis* and *F*. *cancrivora*, respectively. (**c**) The dN/dS of differentially expressed genes in kidney of *F. cancrivora* compared to *F*. *limnocharis*. Red (*F*. *cancrivora*) and blue (*F*. *limnocharis*) bars denote dN/dS of differentially expressed genes, and grey (*F*. *cancrivora*) and pink (*F*. *limnocharis*) bars give dN/dS of background genes. (**d**) Venn diagram of up-regulated genes in kidney, ventral skin and positively selected genes in *F. cancrivora* compared to *F. limnocharis*.

Analyses of orthologous genes suggested rapid evolution occurred in genes potentially involved in the adaptation to seawater. Among the 8698 predicted one-to-one orthologous gene pairs among the three species of frogs, 7173 were annotated as coding genes of *X. tropicalis*. After sequence alignment and trimming, 6959 high-confidence orthologous genes remained for estimating the evolutionary constraints acting on *F. cancrivora*, *F. limnocharis* and *H. rugulosus* (Fig. 2a)^17^,^18^,^19^. We calculated the dN/dS value for each GO term in each branch by the free ratio model implemented in *PAML4*^24^. In total, 54 GO categories harbored significantly higher dN/dS values in *F*. *cancrivora* than in *F*. *limnocharis* (*P* < 0.05, binomial test). These included transmembrane transport (GO:0055085, *P* = 2.31e-32), transporter activity (GO:0005215, *P* = 7.05e-14), transport (GO:0006810, *P* = 6.45e-08), cation transport (GO:0006812, *P* = 1.84e-04), cellular calcium ion homeostasis (GO:0006874, *P* = 3.38e-4), blood vessel development (GO:0001568, *P* = 0.002), electron carrier activity (GO:0009055, *P* = 0.004), sodium ion transport (GO:0006814, *P* = 0.004) and ion transport (GO:0006811, *P* = 0.009) (Fig. 2b and Dataset 5). In contrast, 62 GO categories exhibited accelerated evolutionary rates in *F*. *limnocharis* and these mainly involved metabolism, such as fatty acid metabolic process (GO:0006631, *P* = 1.19e-10), fatty acid beta-oxidation (GO:0006635, *P* = 1.39e-06), triglyceride biosynthetic process (GO:0019432, *P* = 4.83e-05) and fatty acid biosynthetic process (GO:0006633, *P* = 9.28e-05) (Dataset 5).

### Positive selection in *F*. *cancrivora*

The branch-site model executed in *PAML4*^24^ identified 186 candidate positively selected genes among the 6959 orthologous genes (2.7%) (Dataset 6) that possibly associated with the adaptation to high salinity. *ANPEP* and *AVPR2* were significantly enriched in the negative regulation of renal sodium excretion (*P* = 2.41E-03, FDR) (Table 1 and Dataset 7). *ANPEP* encodes aminopeptidase N (CD13) and *AVPR2* encodes the type 2 vasopressin receptor. Further, positively selected sites Q257S in *ANPEP* (sites 73–468, Peptidase_M1_N, IPR014782) and G178N in *AVPR2* (sites1–202, GPCR_Rhodpsn_7TM, IPR017452) were located in conserved domains. Candidate genes *ATP6V1F*, *ATP6V1G3* and *ATP5A*, which are involved in hydrogen-exporting ATPase activity (*P* =  3.707e-02, FDR), were also enriched significantly. The two v-type-H^+^-ATPase enzymes (Table 1) might be associated with the adaptation to high salinity.

**Figure 3:**
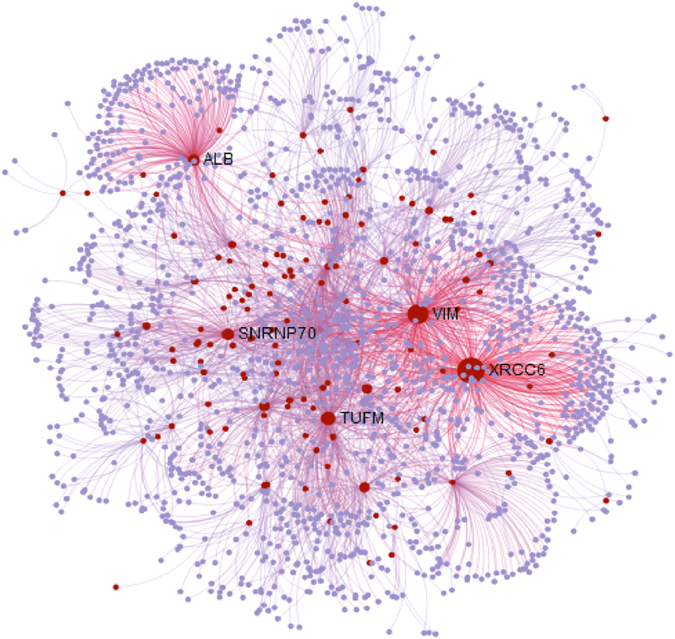
Protein-Protein Interaction network analyses of candidate genes in *Fejervarya cancrivora*. Red dots denote candidate positively selected genes and grey dots represent background genes. The candidate genes with degree ≥ 100 were named.

We mapped the candidate positively selected genes to the protein-protein interaction network database (InnateDB)^25^ to understand their biological functions. Sub-networks of more than five nodes were retained. In total, 129 seed proteins (queries) (69.4.5%) mapped to a sub-network consisting of 1756 nodes (proteins) and 2611 edges (protein-protein interactions). Positively selected *XRCC6*, *VIM*, *TUFM*, *ALB* and *SNRNP70* had the strong ability to interact with other positively selected genes and non-seed proteins (degrees > 100) (Fig. 3). The protein encoded by *ALB* mainly functioned in the regulation of the colloidal osmotic pressure of blood; it may have played an important role in the plasma osmotic pressure of *F. cancrivora*.

### Evolution of gene expression in *F*. *cancrivora*

**Figure 4:**
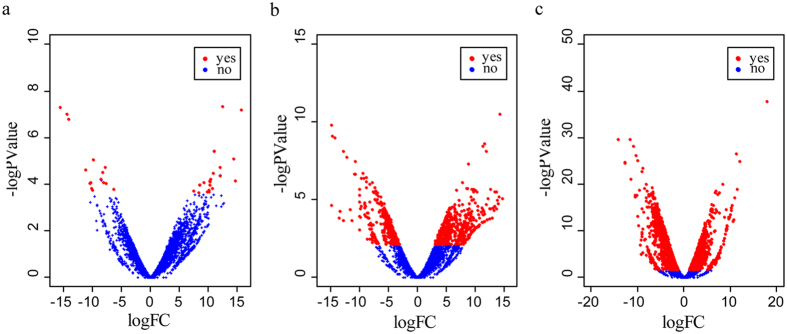
Visualization of differentially expressed genes in brain (a), ventral skin (b) and kidney (c) between *Fejervarya cancrivora* and *F*. *limnocharis.* X-axis represents differential folds of genes (log FC) and the Y-axis shows -log P value of differentially expressed genes. Red denotes significantly up- and down-regulated genes in *F. cancrivora* compared to *F*. *limnocharis*. Blue denotes show genes without significant differences in levels of expressions.

Because changes in gene expression have been considered to play more important roles in phenotypic evolution than mutations in protein-coding sequences^26^, we identified differentially expressed genes between *F. cancrivora* and its closely related species *F. limnocharis*. Kidney harbored far more differentially expressed genes (3239) (Dataset 8) than ventral skin (1104) (Dataset 9) and brain (35) (Fig. 4 and Dataset 10). In *F. cancrivora* the dN/dS values of differentially expressed genes in kidney were significantly higher than those of background genes (*P* = 0.0019, Mann-Whitney *U* test), but this pattern was not found in *F. limnocharis* (*P* = 0.5869, Mann-Whitney *U* test). This discovery suggested that the differentially expressed genes in *F. cancrivora* evolved rapidly (Fig. 2c). Further, in *F. cancrivora* a higher number of positively selected genes were found to harbor elevated expressions in kidney (39 positively selected genes) compared with ventral skin (24 positively selected genes) and brain (1 positively selected gene). Gene enrichment analysis of up-regulated genes in kidney found categories such as transport, regulation of urine volume, regulation of renal sodium excretion, regulation of excretion, sodium ion homeostasis and regulation of vasodilation to be over-represented (Dataset 11). Although this might be associated with adaptation to the high salinity of sea-water, the exact functions of changes in expression remained largely unclear and in need of experimental verification. Consistently, up-regulated genes in the ventral skin also showed similar enrichment. In contrast, down-regulated genes generally were involved in macromolecule metabolic processes and macromolecule modification (Dataset 12).

**Figure 5:**
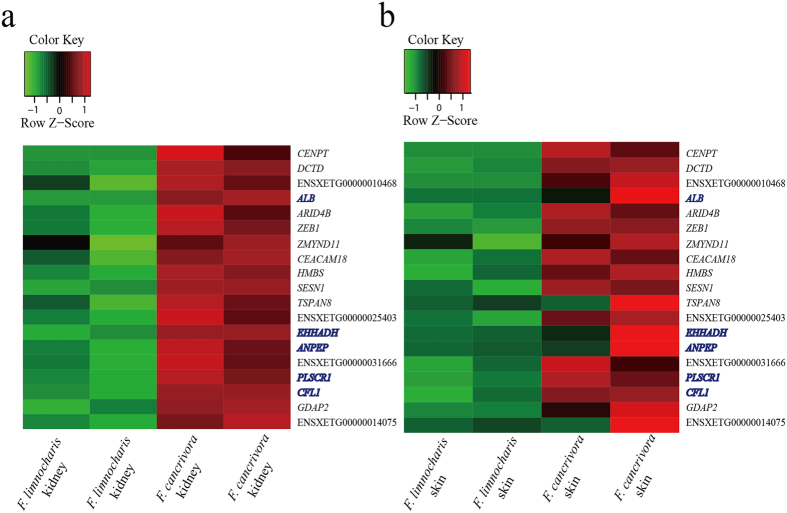
Expression profiles of 19 positively selected and significantly up-regulated genes in kidney (a) and in skin (b) of *Fejervarya cancrivora* compared to *F*. *limnocharis*. Heatmaps show distinct expression profiles of all 19 candidate genes in kidney and ventral skin. The *fpkm* values were normalized by log_2_ (fpkm+1) and each row represents a differentially expressed gene. Blue highlights *ALB*, *EHHADH*, *CFL1*and *PLSCR1*, which associate with regulation of body fluid levels, and *ANPEP*, which associates with negative regulation of renal sodium excretion.

The 19 positively selected genes that exhibited significantly increased levels of expression in the ventral skin and kidney of *F. cancrivora* were also screened (Figs 2d and 5). Genes *ALB*, *EHHADH*, *CFL1*and *PLSCR1* involved in the regulation of body fluid levels exhibited significant enrichment (*P* = 0.0122, FDR) (Dataset 13). In addition, the renin-angiotensin system has long been regarded as an important regulator of systemic blood pressure and renal electrolyte homeostasis and generates a family of bioactive angiotensin peptides with varying biological activities^27^,^28^. Here, *AGT*, *PREP*, *ANPEP* and *CTSA* were found to be up-regulated in the crab-eating frog and enriched significantly in the renin-angiotensin system pathway (*P* = 0.0051, FDR). Further, *NR3C2*, *PIK3CG*, *PDPK1* and *SCNN1G* showed significant up-regulation and they were included in the aldosterone-regulated sodium reabsorption pathway, which was a downstream target pathway of the renin-angiotensin system.

## Discussion

All findings for differentially expressed genes indicate that kidney might contribute more to salt adaptation than the other tested tissues. This discovery is congruent with a previous study^26^ in that overall differentially expressed genes may play a larger role than positively selected genes in adaptations.

Both positively selected and differentially expressed genes exhibit enrichment in the regulation of renal sodium excretion pathway. Renal sodium excretion was found to influence arterial blood pressure by affecting blood volume and electrolyte composition; inversely, blood pressure was coupled to sodium excretion through negative-feedback regulation by the renin-angiotensin-aldosterone system^29^. In this category, *ANPEP* appears to have evolved under positive selection and even experienced a significantly increased level of expression in *F*. *cancrivora*. The gene encodes aminopeptidase N (CD13), a membrane-bound protein that catalyzes the formation of natriuretic hexapeptide angiotensin IV (ANG IV) from ANG III. It was reported to be a candidate gene for handling renal salt in *Rattus norvegicus*^30^,^31^,^32^. Thus, this gene may play a primary role in the tolerance of high salinity by *F*. *cancrivora*. Candidate *AVPR2* appears to have evolved under positive selection. It encodes the type 2 vasopressin receptor, which is a member of the vasopressin/oxytocin receptor subfamily of G protein-coupled receptors^33^. Disruption of *AVPR2* causes X-linked congenital nephrogenic diabetes insipidus (NDI)^34^,^35^. Further, NDI can prevent the concentration of urine after the administration of the antidiuretic hormone arginine-vasopressin^34^. Because hormonal systems were shown to make important contributions to the regulation of osmotic equilibrium in amphibians^13^, positive selection on this gene may drive functional changes in excretion. More importantly, significantly positively selected sites of the two positively selected genes fall precisely into conserved domains. This also implies important functional changes for their protein-activities. Functional experiments can document their importance.

*ATP6V1F* and *ATP6V1G3* associate with ion transport, and in particular with hydrogen-exporting ATPase activity. Functionally, they mediate sodium transport in the mitochondria-rich (MR) cells of amphibian skin as an energizer of Na^+^ uptake. They are considered essential in the maintenance of amphibian body salt^11^,^36^. Thus, the evidence of positive selection on these genes suggests that the crab-eating frog possesses strong if not enhanced capacities for ion transport.

Analyses do not detect an increase in expression for 186 positively selected genes in brain, ventral skin and kidney of *F. cancrivora* relative to closely related *F. limnocharis*. This observation suggests that only a few up-regulated positively selected genes may play key roles in the adaptation of *F*. *cancrivora* to salinity. A group of associated up-regulated positively selected genes involve the osmotic organs kidney and ventral skin. The regulation of body fluid levels involves *ALB*, *EHHADH*, *CFL1* and *PLSCR1*. They show variation in their protein-coding sequences and also harbor strong evidence of up-regulated expression. In particular, *ALB* encodes serum albumin, a major plasma protein that regulates osmotic pressure of blood plasma. The colloid osmotic properties of the albumin’s facilitate plasma volume expansion^37^,^38^. It appears to be an important hub-gene (degree >100) directing construction of the protein-protein interaction network. Consequently, *ALB* may be involved in the adaptation to high-salinity by *F*. *cancrivora*. *EHHADH* was shown to be highly expressed in proximal tubules and associated with energy supply in reabsorption^39^. Thus, synchronous changes in sequences and expression in genes that control body fluid levels may play extraordinary roles in the regulation of the osmotic pressure of blood plasma and the maintenance of electrolytic homeostasis.

**Figure 6:**
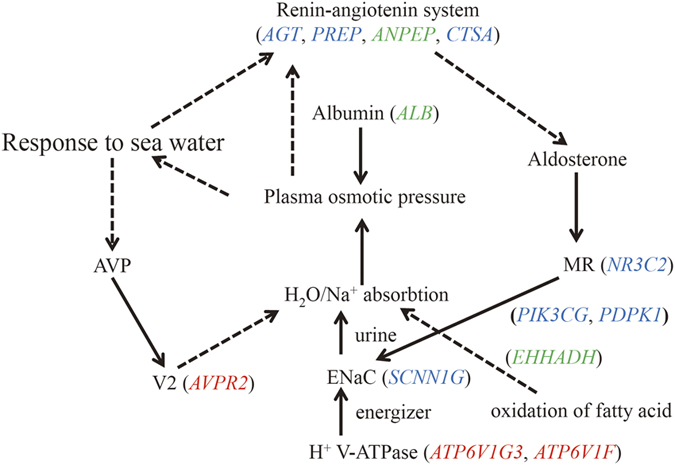
Interactions of positively selected genes and differentially expressed genes involved in the adaptation of *F.* *cancrivora* to high salinity. Red italics denote candidate positively selected genes, blue italics indicate significantly up-regulated genes and green italics genes experiencing positive selection and increased expression. (1) *AVPR2*, expressed mainly in kidney tubules, primarily serves to respond to the pituitary hormone arginine vasopressin (AVP) to maintain water homeostasis; production of *ALB* can facilitate blood plasma volume expansion, which serves to regulate blood pressure. (2) *AGT*, *PREP*, *ANPEP* and *CTSA* play roles in the renin-angiotenin system pathway; *NR3C2*, *PIK3CG*, *PDPK1* and *SCNN1G* associate with aldosterone-regulated sodium reabsorption pathway. (3) *EHHADH* provides energy for tubule reabsorption through the oxidation of fatty acids in the proximal tubule; *ATP6V1G3* and *ATP6V1F* are v-type-H^+^ ATPases and they participate in Na^+^ uptake as energizers.

The renin-angiotensin-aldosterone system plays a primary role in the hormonal osmoregulation of amphibians^13^. In expression levels, except for *ANPEP* with its changes in sequence and expression, *AGT*, *PREP* and *CTSA* also exhibit significant enrichment and markedly higher levels of expressions. Among these genes, *AGT* encodes the precursor protein angiotensinogen^40^, *CTSA* encodes a protein involved in the conversion of angiotensin I to angiotensin II^41^ and *PREP* plays a role in the degradation of angiotensin I and II^42^. Thus, they are important candidate genes for participating in the maintenance of osmotic equilibrium. Importantly, *NR3C2*, *PIK3CG*, *PDPK1* and *SCNN1G*, which associate with the aldosterone-regulated sodium reabsorption pathway as the downstream target pathway of renin-angiotensin system pathway, also possess significantly elevated levels of gene expression. In particular, *NR3C2* encodes MR (mineralocorticoid receptor), which can be activated by mineralocorticoid-aldosterone to regulate Na^+^ homeostasis, largely through multiple mechanisms that modulate the activity of the epithelial Na^+^ channel (ENaC)^43^. Thus, the candidate genes of both pathways plus the candidate positively selected genes (Fig. 6) very likely constitute adaptations of crab-eating frog to their hypertonic environment. Further functional experimentation is necessary to verify this implication.

In summary, our analyses discover positively selected and differentially expressed genes. Both variation in coding sequence and gene expression probably contribute to the adaptation of crab-eating frogs to their saltwater environment. The candidate genes and variants are promising for future studies on high salinity adaptation in poikilothermic animals. Investigations into the functions of these genes and mechanism underlying associated pathways, such as regulation of renal sodium excretion, will make valuable contributions to understanding the system.

## Materials and Methods

The methods were carried out in accordance with approved guidelines.

### Sample collection and RNA isolations of these tissues

Care and treatment of the frogs complied with the guidelines for the National Care and Use of Animals approved by the National Animal Research Authority (P.R. China). The experimental protocols involving live animals were approved by the Ethics and Experimental Animal Committee of Kunming Institute of Zoology, Chinese Academy of Science, China (Approval ID: SYDW-2014013).

*Fejervarya cancrivora* and *F. limnocharis* were collected in mangrove swamps, flooded rice paddies or roadside ditches in Hainan, China. *Hoplobatrachus rugulosus* was acquired in Guangxi, China. Frogs were euthanized by using an intraperitoneal injection of sodium pentobarbital at a dosage of 100 mg per kg body weight. After euthanasia, their brains, kidneys and ventral skin were biopsied quickly, placed in liquid nitrogen, and later stored at −80 °C upon return to the laboratory. Total RNA of these tissues was extracted using TRIzol reagent (Invitrogen Corp., Carlsbad, CA). RNA purifications were performed using an RNeasy Mini Kit (Qiagen, Chatsworth, CA).

### Preparation and sequencing of sample libraries and quality control of transcriptomes

Library constructions from *F. cancrivora* (two biological replicates), *F. limnocharis* (two biological replicates) and *H. rugulosus* (no replicates) were made using Illumina Hiseq2000 RNA sample preparation kits (Illumina, San Diego, CA). After preparing the sequencing libraries from brain, kidney and ventral skin tissues from all three species, we used a 2100-Bioanlyzer (Agilent Technologies) to assess their quality. The libraries were then sequenced on an Illumina Hiseq2000 instrument (Illumina, Inc.) as 2*101 bp. To ensure quality of sequencing, analyses used three lanes for all 15 libraries. The sequencing reads were submitted through the NCBI SRA database and can be accessed via NCBI BioProject accession SRP045588. Sequencing adaptors used for cDNA library construction were trimmed using *Cutadapt* (version_1.2.1)^44^. We employed *Btrim64* (version_0.1.0)^45^ to delete regions with average quality scores of less than 20 and impose a minimal length equal to or greater than 20 bp. The cleaned reads were applied to *de novo* assembly using *Trinity*^46^ (version_2013_08_14) and its default parameters.

### Annotation of transcripts and prediction of open reading frames

First, we used *Blastx*^47^ with parameters, -evalue 1e-5 and -max_target_seqs 1, (version_ ncbi-blast-2.2.27+) to align *de novo* contigs against the protein database of *Nanorana parkeri* (Anuran: Dicroglossidae)^22^ and protein database of *Xenopus tropicalis* (ftp://ftp.ensembl.org/pub/release-75/fasta/xenopus_tropicalis/pep/) to obtain a best hit (BH) of sequence alignment and to produce reliable annotations of the transcriptomes. Because the protein-coding databases of *X. tropicalis* and *N. parkeri* did not include mitochondrial genes, downstream analyses were based on nuclear coding genes only. Next, coding sequences of *F. cancrivora*, *F. limnocharis* and *H. rugulosus* were predicted based on the assumption that the longest open reading frame in the longest transcript per gene had a greatest chance of being a protein-coding region. *GETORF* in *EMBOSS*^48^ (version_6.4.0) was applied to obtain the nucleic sequences between start and stop codons and to limit the minimum size of a fragment to 120 bp. Next, *transeq* in *EMBOSS*^48^ (version_6.4.0) was used to obtain translated protein sequences. The longest protein sequences per gene in three species of frogs were used to identify orthologous genes. We used *OrthoMCL*^49^ (version_2.0.9) to identify orthologous genes using a Markov Cluster algorithm. Downstream analyses used orthologous genes predicted and well annotated for *X. tropicalis* only.

### Calculation of evolutionary rate

We used the dN/dS ratio to measure the evolutionary rate along a lineage. The values of dN, dS, and dN/dS ratio were estimated using the free-ratio model (Parameters: model = 1, NSsites = 0, fix_omega = 0, omega = 1) in *PAML4*^24^ for each branch. Lineage-specific mean values were estimated by concatenated alignments from all orthologs. GO term data were downloaded from BioMart (Ensembl, JGI_4.2.75), and only GO categories with more than 20 orthologs were included in our analyses. GO terms experiencing relatively accelerated evolution were identified using a binomial test^50^.

### Identification of positively selected genes

Orthologous genes were aligned by *PRANK*^51^ (Parameters: -f = fasta -F -codon -noxml -notree -nopost). We aligned the longest predicted ORFs for the longest transcript-pairs across the three species. *Gblocks*^52^,^53^ (version_0.91b; Parameters: -t = c -b3 = 1 -b4 = 6 -b5 = n) was employed to reduce the rate of false positive predictions due to potential sequencing errors, incorrect alignments and non-orthologous regions based on codons. After trimming, we removed fragment lengths shorter than 100 bp to obtain one-to-one orthologous genes. We calculated the ratio of nonsynonymous (Ka or dN) to synonymous (Ks or dS) substitution rate (ω = Ka/Ks or dN/dS) using *CODEML* in the *PAML4* package^24^. The accepted species-tree ((*F. cancrivora*, *F. limnocharis*), *H. rugulosus*) was used as the guide tree^17^,^18^,^19^. Branch-site model^24^ (Parameters: Null hypothesis: model = 2, NSsites = 2, fix_omega = 1, omega = 1; Alternative hypothesis: model = 2, NSsites = 2, fix_omega = 0, omega = 1) was used to detect positively selected genes. A likelihood ratio test compared the alternative hypothesis of positive selection on the foreground branch to a null hypothesis with no positive selection on the background branch for each orthologous gene. Positively selected genes were inferred only if their *P* values were less than 0.05. Positively selected sites were deduced by Bayes Empirical Bayes (BEB) analyses^54^. Functionally conserved domains of positively selected genes were predicted by *InterProsan*^55^ (version_5.2-45.0).

### Detection of differentially expressed genes between *F. cancrivora* and *F. limnocharis*

*RSEM*^56^ (version_1.2.3), an accurate transcript quantification tool for RNA-Seq data with or without a reference genome, was utilized to quantify the transcripts. Briefly, we viewed the longest orthologous transcript sequences per species of frog as the reference databases and then applied rsem-calculate-expression to map trimmed pair-end reads of brain, kidney and ventral skin onto it. For the same tissues, we used the output of *RSEM* as input for *EdgeR*^57^, an R software library of bioconductor (http://www.bioconductor.org), to detect differentially expressed genes between *F. cancrivora* and *F. limnocharis*. Analyses considered genes whose false discovery rate (FDR) values, using the BH approach^58^, were less than or equal to 0.05 to be differentially expressed. The heat maps were drawn as follows: function “heatmap.2” of the “gplots” library of R package.

### Gene Ontology enrichment analyses

We identified GO categories at three levels (biological process, molecular function and cellular component) and pathways that were over-represented by positively selected genes and differentially expressed genes using the *MetaCore*^59^ pipeline. Enrichment *P*-values were calculated. All *P*-values were multi-normalized FDR using the BH approach^58^. Categories with a FDR of less than 0.05 were considered over-represented.

### Protein-Protein Interaction (PPI) Network

We mapped the candidate positively selected genes to protein-protein interaction network database (InnateDB). Sub-network topologies were obtained by *NetworkAnalyst* (http://www.networkanalyst.ca/NetworkAnalyst)^60^. The sub-networks with node counts ≤5 were filtered and statistics of the total nodes, edges and seed proteins were obtained from the mapping overview.

## Additional Information

**How to cite this article**: Shao, Y. *et al.* Transcriptomes reveal the genetic mechanisms underlying ionic regulatory adaptations to salt in the crab-eating frog. *Sci. Rep.* **5**, 17551; doi: 10.1038/srep17551 (2015).

## Supplementary Material

Supplementary Dataset 1

Supplementary Dataset 2

Supplementary Dataset 3

Supplementary Dataset 4

Supplementary Dataset 5

Supplementary Dataset 6

Supplementary Dataset 7

Supplementary Dataset 8

Supplementary Dataset 9

Supplementary Dataset 10

Supplementary Dataset 11

Supplementary Dataset 12

Supplementary Dataset 13

## Figures and Tables

**Table 1:** Positively selected genes involved in renal sodium excretion and H^+^ATPase in *F. cancrivora*.

Gene name	Description	*P*-value
*ANPEP*	Alanyl (membrane) aminopeptidase	0.0002
*AVPR2*	Arginine vasopressin receptor 2	0.0058
*ATP6V1F*	ATPase, H+ transporting, lysosomal 14kDa, V1 subunit F	0.0107
*ATP6V1G3*	ATPase, H+ transporting, lysosomal 13kDa, V1 subunit G3	0.0279
